# Distinct Time Course of the Decrease in Hepatic AMP-Activated Protein Kinase and Akt Phosphorylation in Mice Fed a High Fat Diet

**DOI:** 10.1371/journal.pone.0135554

**Published:** 2015-08-12

**Authors:** Mami Shiwa, Masayasu Yoneda, Hirofumi Okubo, Haruya Ohno, Kazuhiro Kobuke, Yuko Monzen, Rui Kishimoto, Yusuke Nakatsu, Tomoichiro Asano, Nobuoki Kohno

**Affiliations:** 1 Department of Molecular and Internal Medicine, Graduate School of Biomedical and Health Sciences, Hiroshima University, Hiroshima, Japan; 2 Department of Biomedical Chemistry, Graduate School of Biomedical and Health Sciences, Hiroshima University, Hiroshima, Japan; East Tennessee State University, UNITED STATES

## Abstract

AMP-activated protein kinase (AMPK) plays an important role in insulin resistance, which is characterized by the impairment of the insulin-Akt signaling pathway. However, the time course of the decrease in AMPK and Akt phosphorylation in the liver during the development of obesity and insulin resistance caused by feeding a high fat diet (HFD) remains controversial. Moreover, it is unclear whether the impairment of AMPK and Akt signaling pathways is reversible when changing from a HFD to a standard diet (SD). Male ddY mice were fed the SD or HFD for 3 to 28 days, or fed the HFD for 14 days, followed by the SD for 14 days. We examined the time course of the expression and phosphorylation levels of AMPK and Akt in the liver by immunoblotting. After 3 days of feeding on the HFD, mice gained body weight, resulting in an increased oil red O staining, indicative of hepatic lipid accumulation, and significantly decreased AMPK phosphorylation, in comparison with mice fed the SD. After 14 days on the HFD, systemic insulin resistance occurred and Akt phosphorylation significantly decreased. Subsequently, a change from the HFD to SD for 3 days, after 14 days on the HFD, ameliorated the impairment of AMPK and Akt phosphorylation and systemic insulin resistance. Our findings indicate that AMPK phosphorylation decreases early upon feeding a HFD and emphasizes the importance of prompt lifestyle modification for decreasing the risk of developing diabetes.

## Introduction

Insulin resistance is characterized by impaired insulin signaling, which is a precursor to type 2 diabetes. A high fat diet (HFD) interferes with the phosphorylation of Akt, a serine/threonine-specific protein kinase that plays a central role in insulin signaling [1] via inhibition of gluconeogenesis in the liver [2] and glucose uptake in skeletal muscle [3], resulting in hyperglycemia.

AMP-activated protein kinase (AMPK) is phosphorylated and activated in response to an increased cellular AMP/ATP ratio induced by starvation or exercise, thereby acting as an energy sensor that regulates cellular metabolism [4]. Phosphorylated AMPK suppresses gluconeogenesis in the liver [5] and increases fatty acid oxidation in the liver and skeletal muscle [6,7]. Additionally, increased AMPK activity promotes glucose uptake into skeletal muscle [6,8]. Metformin is an established treatment for type 2 diabetes owing to its ability to increase peripheral glucose uptake and reduce hepatic glucose production (HGP) in an AMPK-dependent manner [9,10]. Although AMPK signaling has been highlighted as an important pathway associated with insulin resistance independent of the insulin-Akt signaling pathway, no studies have been conducted on the synchronous time course of impaired Akt and AMPK phosphorylation due to a HFD.

It is well established that moderate weight reduction can improve insulin resistance, impaired glucose tolerance, and dyslipidemia in obese and diabetic subjects [11–14]. These findings suggest that intracellular lipids in the liver and skeletal muscle are associated with insulin resistance and that diet modification reduces intrahepatic lipid levels 1‒2 weeks before a decrease in body weight or intramyocellular lipid level are detected [12,13]. However, it is unclear when Akt and AMPK phosphorylation are restored during dietary intervention after the development of ectopic lipid accumulation and insulin resistance.

We conducted a detailed time course study by using mice fed a HFD to determine whether a time lag exists between decreased Akt phosphorylation and AMPK phosphorylation in the liver during the development obesity and insulin resistance. Furthermore, we aimed to determine whether a change from a HFD to a standard diet (SD) (hereafter referred to as dietary change) could improve the impairment of these two signaling pathways.

## Materials and Methods

### Animals and diets

All protocols were approved by the institutional review board of Hiroshima University. Six-week-old male ddY mice, purchased from Kyudo Company (Saga, Japan), were fed *ad libitum* and maintained on a 12-hour light/dark cycle under controlled environmental conditions. In the initial experiments, mice were fed a SD (12.7 kcal% fat, 61.6 kcal% carbohydrate, and 25.7 kcal% protein) or a lard-based HFD (62.2 kcal% fat, 19.6 kcal% carbohydrate, and 18.2 kcal% protein) for 28 days. In subsequent experiments, mice were divided into two groups: the SD group and the dietary change group. The latter group was fed the HFD for 14 days, followed by the SD for 14 days. The SD and HFD were purchased from Oriental Yeast (Tokyo, Japan).

### Insulin and glucose tolerance tests

The intraperitoneal insulin tolerance test (ITT) and the glucose tolerance test (GTT) were performed on days 3, 7, 14, 17, or 28. ITT (1 U/kg, Humulin-R, Eli Lilly and Co., Indianapolis, IN, US) was performed after a 4-hour fasting, whereas GTT (1.5 g/kg, D-glucose, Wako, Osaka, Japan) was performed after a 16-hour fasting. Blood glucose level was measured at 0, 30, 60, 90, and 120 min for ITT and at 0, 15, 30, 60, 90, and 120 min for GTT. Serum samples were obtained from the tail vein.

### Western blot assays

All mice were anesthetized with an intraperitoneal injection of pentobarbital sodium (60 mg/kg) after overnight fasting, sacrificed, and liver tissues were collected. For assessing insulin-stimulated Akt phosphorylation, insulin (2 U/kg, Humulin-R) was injected intraperitoneally in mice fasted for 3 hours and liver samples were harvested 10 minutes after insulin injection. Tissues were quickly homogenized in ice-cold lysis buffer (50 mM Tris-HCl [pH 7.5], 150 mM NaCl, 1 mM ethylenediaminetetraacetic acid, 1% Triton X-100, 1 mM sodium orthovanadate, and 1 mM phenylmethylsulfonyl fluoride), and centrifuged at 16,000 × *g* for 30 min at 4°C. The supernatants, which were used as liver lysates, were added to sodium dodecyl sulfate (SDS) sample buffer (200 mM Tris-HCl [pH 6.8], 4% SDS, 10% glycerol, 0.1% bromophenol blue, and 5% 2-mercaptoethanol), boiled for 5 min, and analyzed by western blotting. Antibodies against AMPKα (62 kDa), phosphorylated AMPKα-Thr172 (62 kDa), Akt (60 kDa), phosphorylated Akt-Ser473 (60 kDa), or β-actin (45 kDa) (Cell Signaling Technology, Danvers, MA, US), were used as primary antibodies, followed by incubation with an anti-rabbit horseradish peroxidase-conjugated secondary antibody. The antigen-antibody interactions were visualized using the SuperSignal West Pico Chemiluminescent Substrate (Thermo Fisher Scientific, Waltham, MA, US). Band quantification and analysis was performed separately for each gel with ImageJ software (National Institutes of Health, Bethesda, MD, US). Representative bands from two animals from each group are shown.

### Histological analyses

After 12-hour fasting, liver tissues were harvested, fixed in 4% buffered formalin, and embedded in paraffin. Sections were cut and stained with Oil Red O, and processed at Kyodo Byori Inc. (Kobe, Japan).

### Statistics

Statistical analyses were performed using Student’s *t* test or repeated measures analyses of variance. *P*-values less than 0.05 were considered significant. Values are expressed as mean ± standard deviation.

## Results

### Impacts of short-term HFD on weight gain and liver steatosis

**Fig 1:**
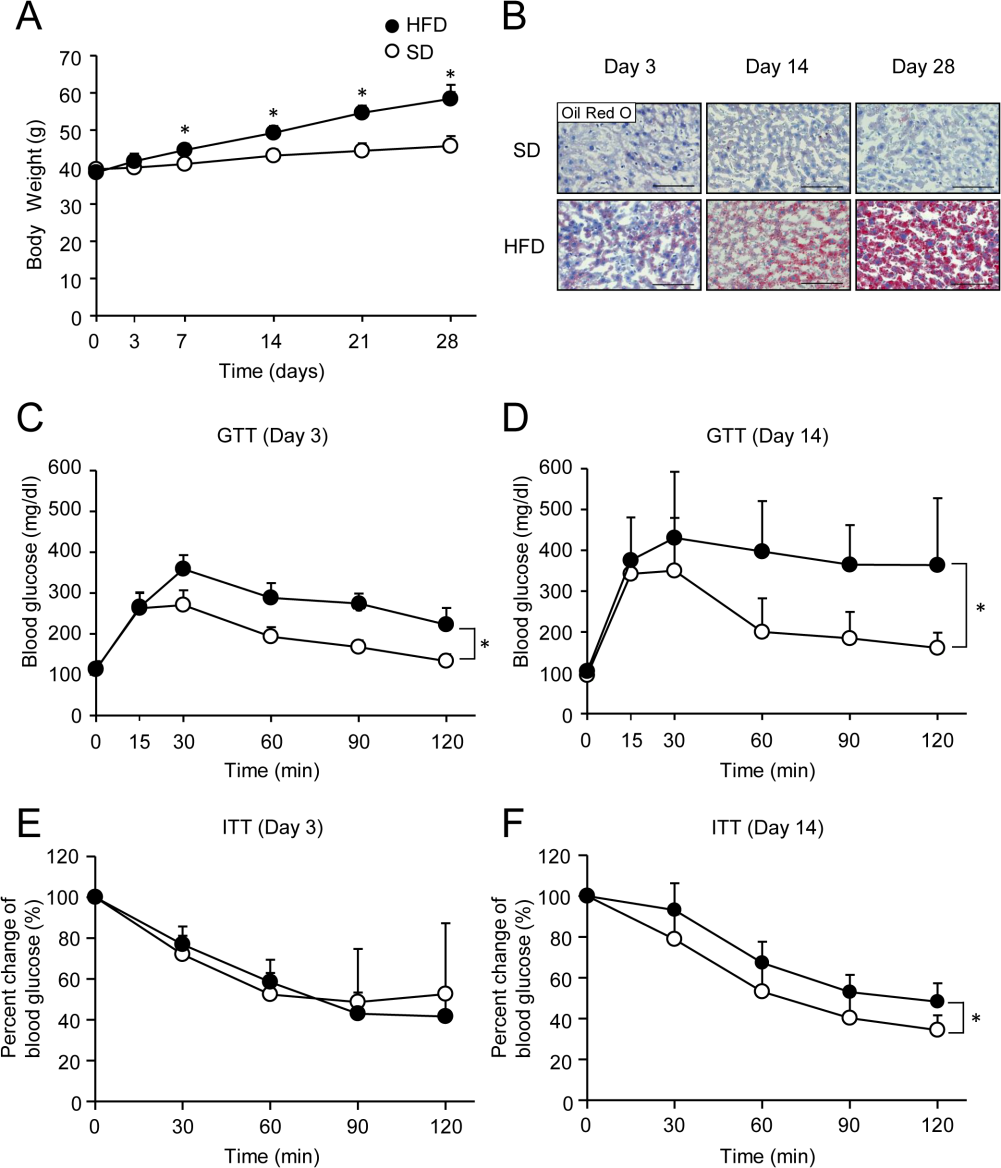
Development of obesity, liver steatosis, glucose intolerance, and insulin resistance in high fat diet-fed mice. (A) Body weight in mice fed a standard diet (SD; white circles) or high fat diet (HFD; black circles). Results are means ± standard deviation. Error bars are smaller than the symbols. n = 5 per group. *P < 0.05. (B) Oil Red O-stained liver sections from SD- or HFD-fed mice. Scale bar, 100 μm. (C and D) Intraperitoneal glucose tolerance test (GTT) in SD- or HFD-fed mice on day 3 (C) or day 14 (D). (E and F) Insulin tolerance test (ITT) in SD- or HFD-fed mice on day 3 (E) or day 14 (F). Results are means ± standard deviation. n = 5‒8 per group. *P < 0.05.

In initial studies, mice were fed a HFD for 3 to 28 days to assess the development of weight gain and liver steatosis. Body weight in mice fed the HFD increased slightly after 3 days and then significantly increased after 7 days when compared with mice fed the SD (Fig 1A). On histological examination, increased oil red O staining indicative of hepatic lipid accumulation was observed in mice fed on the HFD for 3 days and increased on this diet for up to 28 days (Fig 1B). To determine temporal alterations of glucose metabolism and insulin sensitivity by the HFD, GTT and ITT were performed. Glucose intolerance in mice fed the HFD was evident only after 3 days (Fig 1C) and worsened on this diet for up to 14 days (Fig 1D). Insulin-dependent blood glucose reduction was similar between the SD and HFD groups on day 3 (Fig 1E) until day 7 (data not shown). However, a significant difference was observed between the groups on day 14 (Fig 1F), suggesting that systemic insulin resistance occurred between from 7 days to 14 days of HFD feeding.

### Time course of AMPK and Akt phosphorylation levels in livers of mice fed the HFD

**Fig 2:**
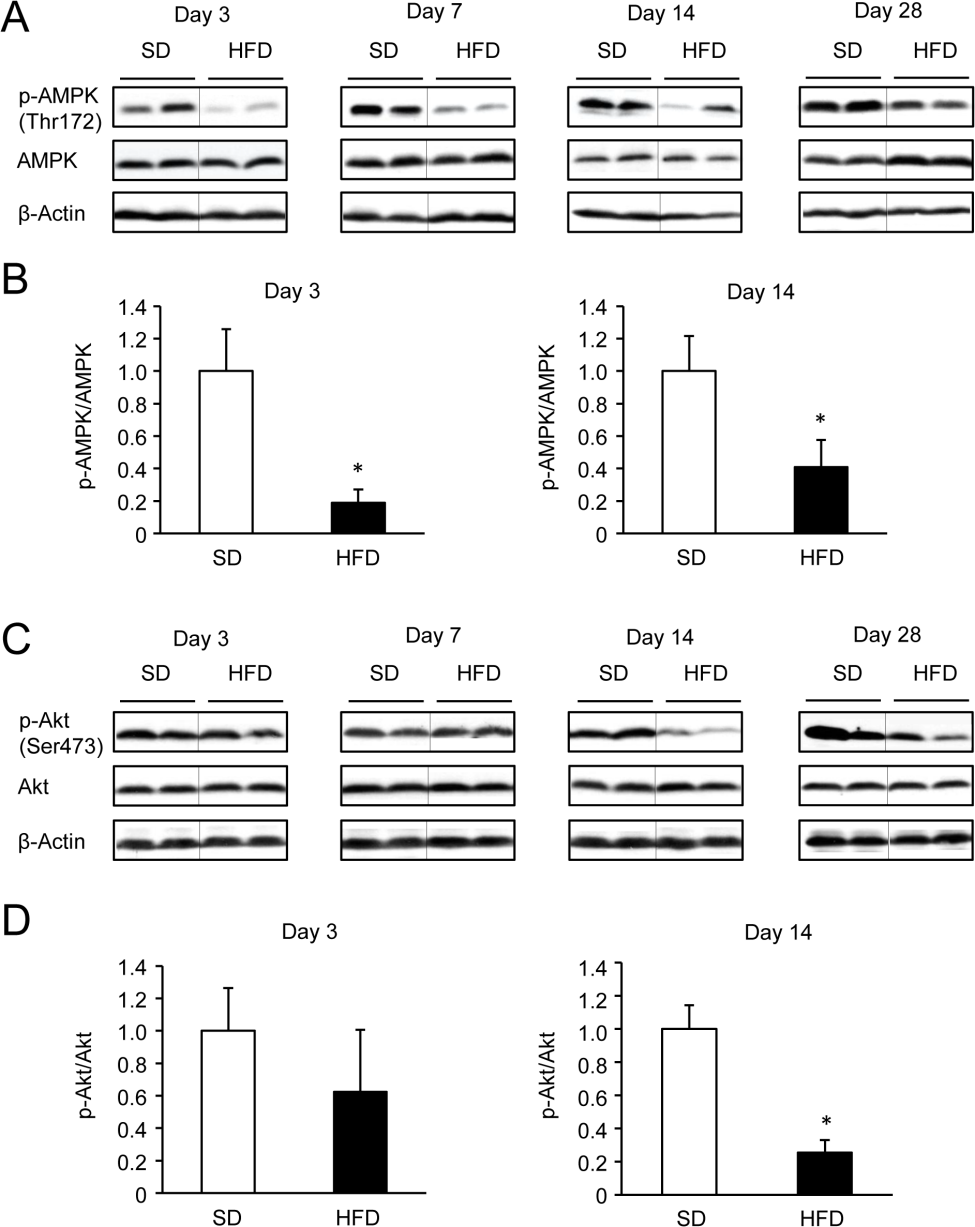
Hepatic AMPK and Akt phosphorylation levels in SD- or HFD-fed mice. (A and B). Representative western blots and quantification of AMPK phosphorylation (relative to total AMPK protein). n = 4 per group. *P < 0.05. (C) and (D). Representative western blots and quantification of insulin-stimulated Akt phosphorylation (relative to total Akt protein). n = 4 per group. *P < 0.05.

To gain insight into the development of the impaired phosphorylation of AMPK and Akt in the liver, protein amounts and phosphorylation levels were compared between SD- and HFD-fed mice using western blotting. AMPK protein levels were not significantly different between the groups. However, phosphorylated AMPK levels were significantly decreased in mice fed the HFD after 3 days and for up to 28 days (Fig 2A and 2B). In contrast, phosphorylated Akt levels were not decreased in mice fed the HFD until day 14 and remained significantly decreased for 28 days (Fig 2C and 2D). These results suggest that a 3-days HFD is enough to suppress hepatic AMPK phosphorylation, while a 14-days HFD is necessary to suppress hepatic Akt phosphorylation.

### Impacts of dietary change on weight gain, liver steatosis, and insulin resistance

**Fig 3:**
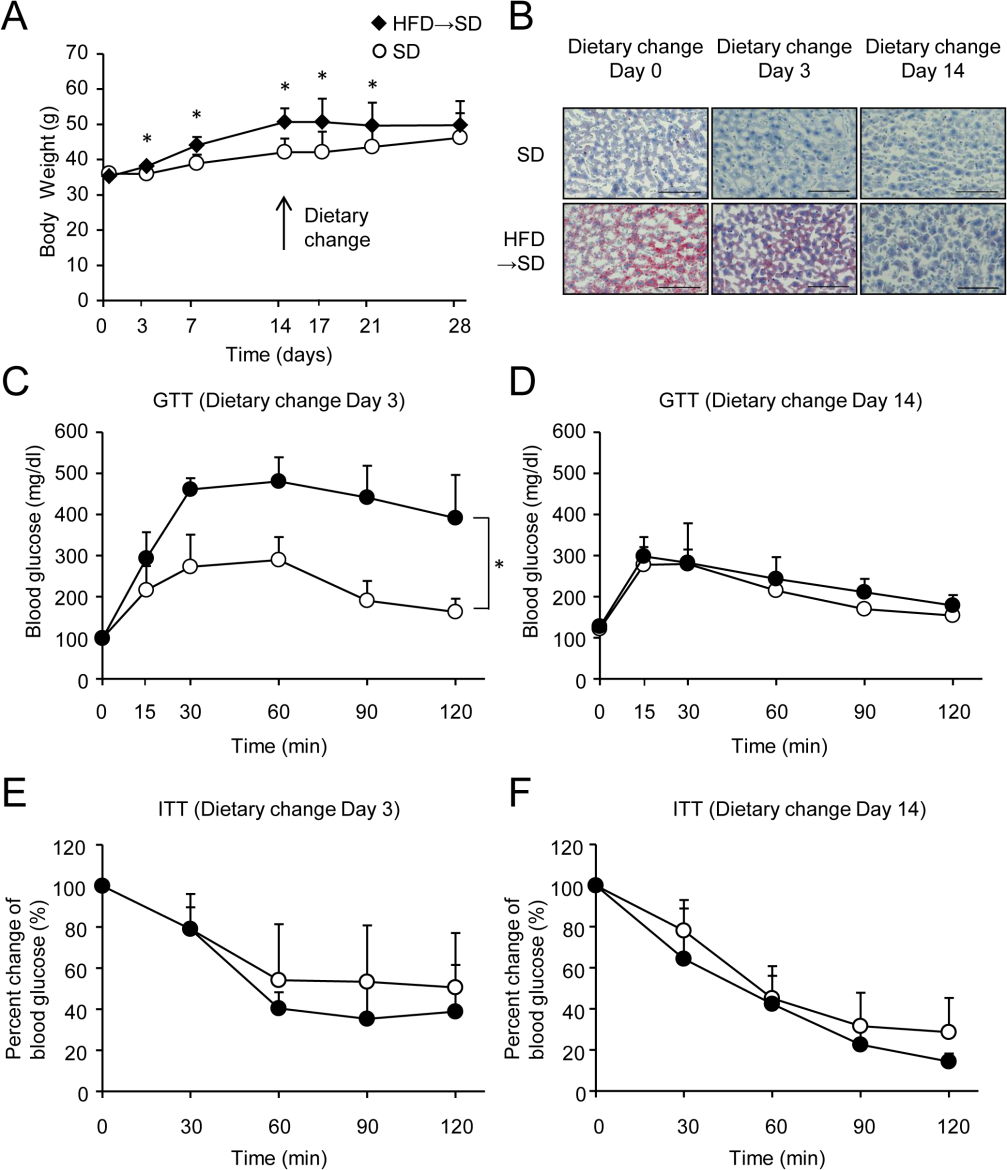
Dietary change improves weight gain, liver steatosis, glucose tolerance, and insulin resistance. The diet was changed on day 14 (black diamonds) or continued as a SD (white circles). (A) Body weight. Results are means ± standard deviation. n = 5 per group. *P < 0.05. (B) Oil Red O-stained liver sections from SD-fed mice or those subjected to a dietary change. Scale bar, 100 μm. (C and D) Intraperitoneal glucose tolerance test (GTT) in SD-fed mice or those subjected to a dietary change on day 3 (C) or day 14 (D). (E and F) Insulin tolerance test (ITT) in SD-fed mice or those subjected to a dietary change on day 3 (E) or day 14 (F). Results are means ± standard deviation. n = 4‒5 per group. *P < 0.05.

To determine the effects of diet modification on HFD-induced weight gain and insulin resistance, mice were fed the HFD for 14 days followed by the SD for 14 days. Changes in body weight, hepatic lipid accumulation, glucose tolerance, and systemic insulin sensitivity were then evaluated in these mice. In this series of experiments, a significant weight gain was observed in mice fed the HFD for 3 days compared with SD mice (Fig 3A). The diet change from the HFD to the SD for 14 days reversed the weight gain (Fig 3A), increased oil red O staining indicative of liver steatosis (Fig 3B), and glucose intolerance caused by the HFD (Fig 3C and 3D). Systemic insulin resistance almost improved 3 days after the dietary change and this improvement was sustained until day 14, as assessed by the ITT (Fig 3E and 3F).

### Time course of AMPK and Akt phosphorylation levels in livers after a dietary change

**Fig 4:**
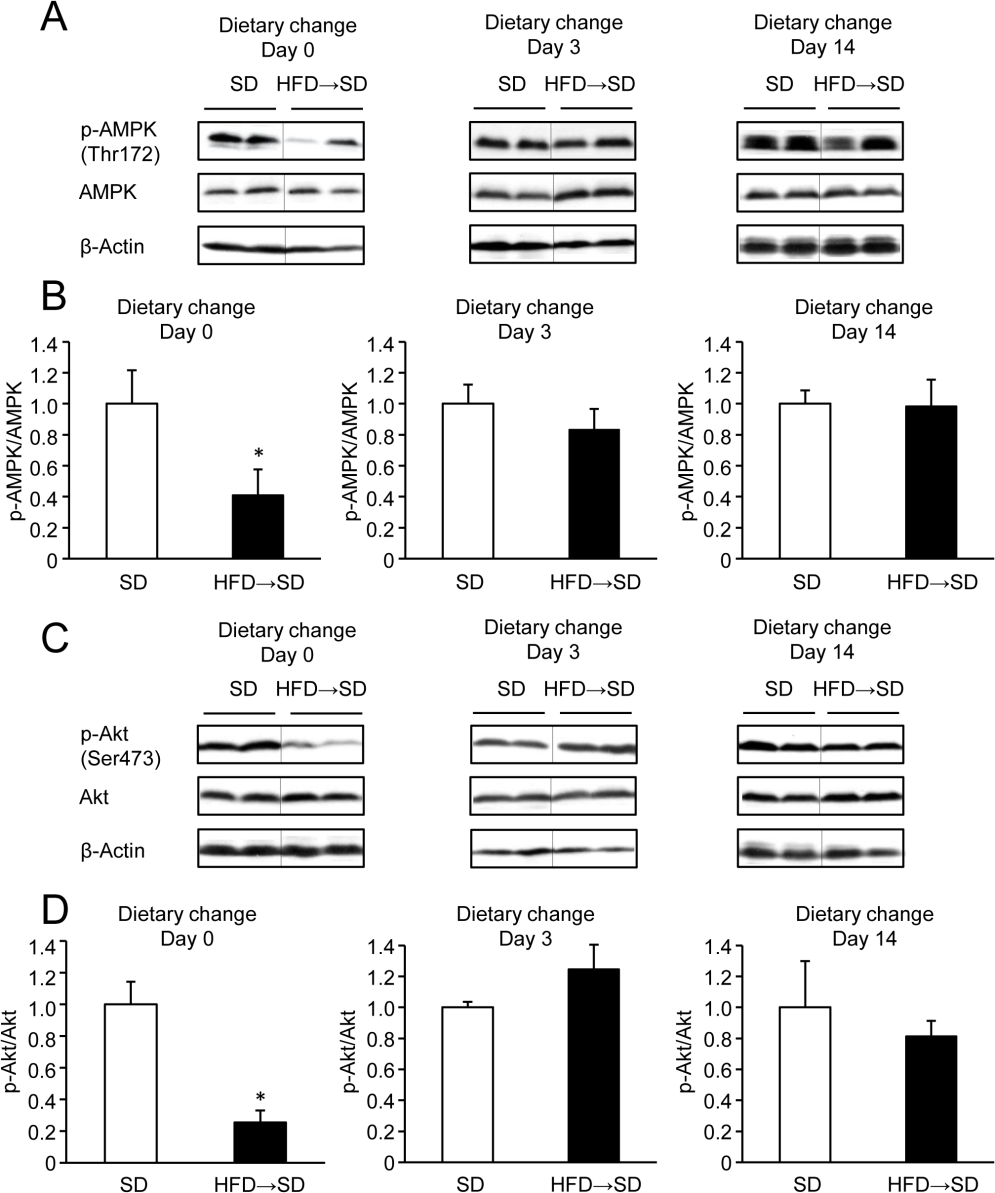
Hepatic AMPK and Akt phosphorylation levels in mice subjected to dietary change or SD. (A and B) Representative western blot quantification of AMPK phosphorylation (relative to total AMPK protein). n = 4‒5 per group. (C and D) Representative western blot quantification of insulin-stimulated Akt phosphorylation (relative to total Akt protein). n = 4‒5 per group.

To investigate whether a diet change from the HFD to the SD reversed the decrease in AMPK and Akt phosphorylation, phosphorylation levels were compared between mice subjected to dietary change and SD. Three days after the dietary change, both phosphorylated AMPK (Fig 4A and 4B) and phosphorylated Akt levels (Fig 4C and 4D) recovered to a level similar to that of the SD group. These results suggest that dietary change improves both AMPK and Akt phosphorylation in the liver within 3 days, independent of obesity and hepatic steatosis.

## Discussion

The present study revealed that body weight and hepatic lipid accumulation increased, and the AMPK phosphorylation levels decreased, early during feeding a HFD. Subsequently, the levels of Akt phosphorylation decreased, coinciding with systemic insulin resistance. Previous reports (S1 Table) have shown that AMPK phosphorylation levels in the livers of rodents decreased after being fed a HFD for a relatively short period (e.g., for 2‒3 weeks) [15,16] or a long period (e.g., 15‒19 weeks) [17–20]. Consistent with previous reports, we detected a decreased level of AMPK phosphorylation and liver steatosis simultaneously. Interestingly, we found that the hepatic AMPK phosphorylation levels decreased in mice fed a HFD for an extremely short period of 3 days.

Our results indicate that the time of occurrence of decreased Akt phosphorylation was distinct from that of decreased AMPK phosphorylation in the liver of mice fed the HFD. Several studies have reported that ingestion of a HFD for only 3 to 4 days induced hepatic triglyceride (TG) accumulation and inflammation, increased HGP during glucose clamp studies, and decreased hepatic Akt phosphorylation [21–24, S2 Table]. These results are inconsistent with our finding that Akt phosphorylation levels decreased after hepatic lipid accumulation. Meanwhile, Turner et al. [25] reported that hepatic TG accumulation and the inability to suppress endogenous glucose production developed 1 week after feeding on a HFD, whereas a decrease in hepatic Akt phosphorylation and inflammation was not induced after 3 weeks on a HFD. Furthermore, Lee et al. [26] found that after 3 days, a HFD induced an increase in basal HGP but not hepatic inflammation which required feeding a HFD for 16 weeks. These results demonstrate that hepatic steatosis and increased HGP develop earlier compared to induction of hepatic inflammation and impaired hepatic Akt phosphorylation during HFD feeding. Taken together, these results suggest the possibility that decreased AMPK phosphorylation led to hepatic lipid accumulation after 3 days of HFD feeding, after which excessive lipid-induced hepatic inflammation may have led to decreased Akt phosphorylation levels after 14 days of HFD. Furthermore, the elevated blood glucose levels observed after glucose loading in the HFD mice on day 3 suggest that hepatic insulin resistance might have developed at that point; however, this mechanism remains a matter of speculation, because we did not evaluate hepatic inflammation or perform euglycemic insulin clamp studies.

The current results show that a diet change from the HFD to the SD reversed the decrease in AMPK and Akt phosphorylation and systemic insulin resistance caused by the HFD, and resulted in improvements in hepatic steatosis and obesity. Dietary intervention for patients with type 2 diabetes also reduces hepatic lipid contents and improves hepatic insulin resistance within 1 to 2 weeks [12,13]. When mice were fed a HFD for 12 weeks, followed by a change to a low fat diet for 3 weeks, hepatic TG accumulation decreased, and hepatic inflammation and insulin resistance were attenuated [27]. These findings suggest that improvements in insulin resistance are associated with a decreased accumulation of hepatic lipids. However, prior studies have not evaluated the phosphorylation levels of AMPK and Akt. We showed that interrupting the lipid overload immediately improved the intracellular energy state and increased AMPK phosphorylation. In addition, an abrupt decrease in the abundance of dietary fatty acids, which directly impair the insulin-Akt signaling pathway [28–30], have rapidly increased Akt phosphorylation. Further studies are required to determine whether recovery from HFD-induced disturbances in cell signaling can occur when the HFD is administered for a longer period, because mice were fed a HFD for only 2 weeks in this study.

It has reported that the AMPK phosphorylation levels decrease in the skeletal muscle of obese rats fed a HFD [31], whereas others studies, including ours (S1 Fig), have found that AMPK phosphorylation levels in the skeletal muscle of mice fed a HFD were similar to those of mice fed a SD [32]. According to studies that examined the time course of hepatic and peripheral insulin resistance in rats fed a HFD, insulin resistance in muscles occurs after the development of hepatic insulin resistance [33,34]. Additionally, these reports support our result that feeding on a HFD for 14 days is insufficient to reduce AMPK phosphorylation in skeletal muscle.

In conclusion, we demonstrate that the hepatic AMPK phosphorylation levels decrease early after initiation of a HFD and are evident before the decrease in Akt phosphorylation and systemic insulin resistance. Furthermore, a diet change from a HFD to a SD rapidly restores AMPK and Akt phosphorylation to normal levels. Moreover, the Diabetes Prevention Program (a large clinical study) has shown that lifestyle changes and metformin treatment reduce the risk of developing diabetes in glucose-intolerant individuals [35]. Our findings highlight the importance of promptly modifying the lifestyle to prevent the development of insulin resistance and diabetes, because hepatic AMPK activity decreases early after the consumption of high-fat foods.

## Supporting Information

S1 Fig Phosphorylated AMPK levels in skeletal muscle. Representative western blots from skeletal muscle lysates of SD- or HFD-fed mice using the indicated antibodies.
(TIF)

S1 Table Summary of research on the decreased phosphorylated AMPK levels in rodents fed a HFD. HFD, high fat diet; TG, triglyceride; ITT, insulin tolerance test; GTT, glucose tolerance test; BAT, brown adipose tissue; WAT, white adipose tissue
(PDF)

S2 Table Summary of research on the decreased phosphorylated Akt levels in rodents fed a HFD. HFD, high fat diet; GIR, glucose infusion rate; HGP, hepatic glucose production; TG, triglyceride; GTT, glucose tolerance test
(PDF)

S3 Table The ARRIVE Guidelines Checklist. (PDF)
