# Supplementary figures and images for: Distinct Time Course of the Decrease in Hepatic AMP-Activated Protein Kinase and Akt Phosphorylation in Mice Fed a High Fat Diet
Source: PLoS One. 2015 Aug 12;10(8):e0135554. doi: 10.1371/journal.pone.0135554 (PMC4534138; doi:10.1371/journal.pone.0135554)

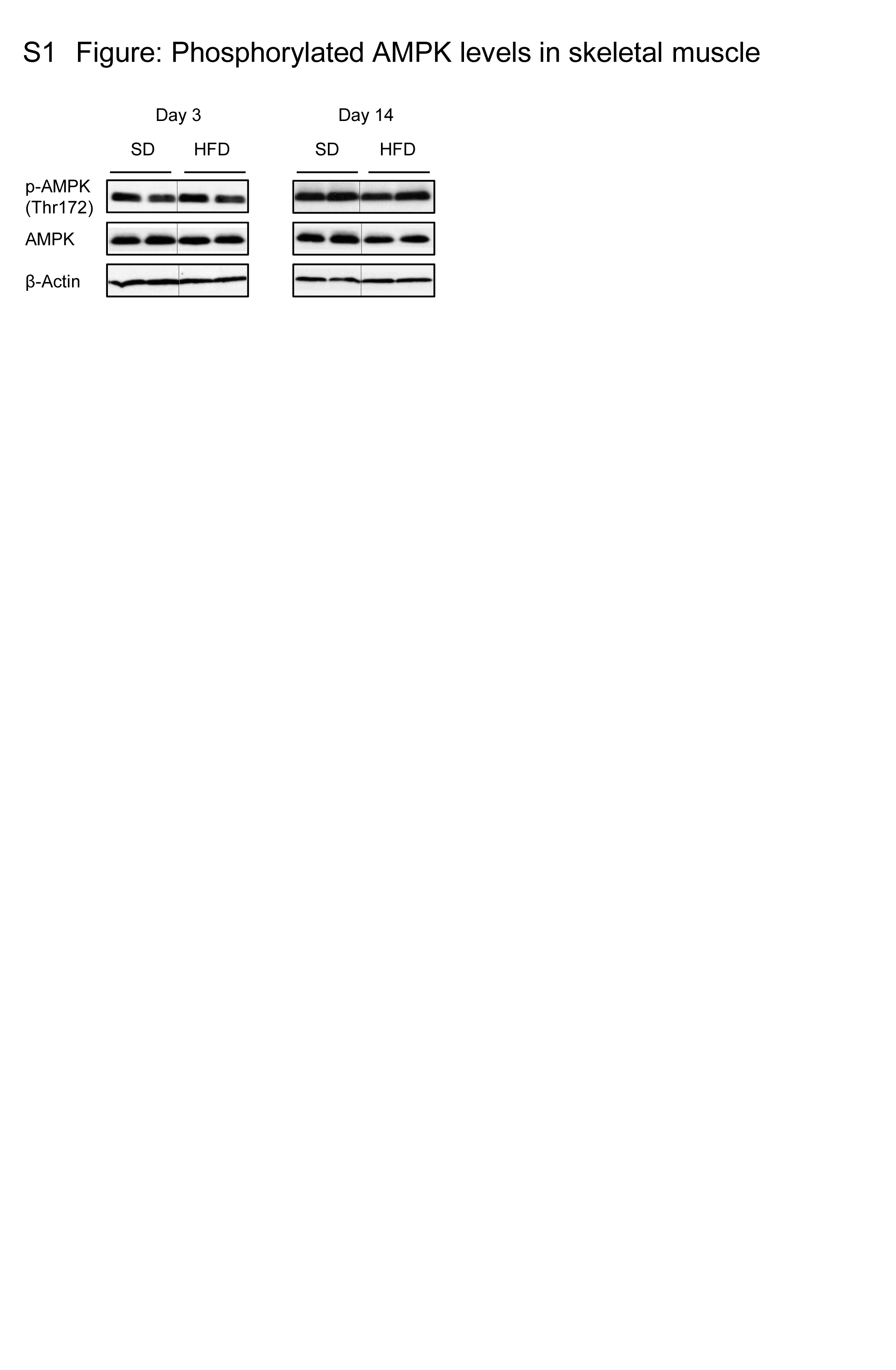

Supplement: S1 Fig — Representative western blots from skeletal muscle lysates of SD- or HFD-fed mice using the indicated antibodies. (TIF) [file pone.0135554.s001.tif]
