# Supplementary material for: Distinct Time Course of the Decrease in Hepatic AMP-Activated Protein Kinase and Akt Phosphorylation in Mice Fed a High Fat Diet
Source: PLoS One. 2015 Aug 12;10(8):e0135554. doi: 10.1371/journal.pone.0135554 (PMC4534138; doi:10.1371/journal.pone.0135554)
Supplement: S1 Table — HFD, high fat diet; TG, triglyceride; ITT, insulin tolerance test; GTT, glucose tolerance test; BAT, brown adipose tissue; WAT, white adipose tissue (PDF) [file pone.0135554.s002.pdf]

S1 Table. Summary of research on the decreased phosphorylated AMPK levels in rodents fed a HFD

Strain	Age	HFD period	Composition	Further characteristics	Refs
ddY mouse	7 weeks	2 weeks	60% fat	No significant increase in body weight and fasting glucose	15
Sprague-Dawley rat	8 weeks	3 weeks	60% fat	No significant increase in body weight and fasting glucose	16
Sprague-Dawley rat	3 weeks	15 weeks	45% fat	Increased fasting glucose and hepatic TG content	17
C57BL/6J mouse	5–6 weeks	16 weeks	60% fat	Insulin resistance in ITT and glucose intolerance in GTT, increased hepatic steatosis	18
Sprague-Dawley rat	8 weeks	18 weeks	60% fat	Increased BAT and epididymal WAT weight	19
Sprague-Dawley rat	7 weeks	19 weeks	45% fat	Increased body weight and fasting glucose	20
