# Supplementary material for: Distinct Time Course of the Decrease in Hepatic AMP-Activated Protein Kinase and Akt Phosphorylation in Mice Fed a High Fat Diet
Source: PLoS One. 2015 Aug 12;10(8):e0135554. doi: 10.1371/journal.pone.0135554 (PMC4534138; doi:10.1371/journal.pone.0135554)
Supplement: S2 Table — HFD, high fat diet; GIR, glucose infusion rate; HGP, hepatic glucose production; TG, triglyceride; GTT, glucose tolerance test (PDF) [file pone.0135554.s003.pdf]

S2 Table. Summary of research on the decreased phosphorylated Akt levels in rodents fed a HFD

Strain	Age	HFD period	Composition	Glucose clamp	Further characteristics	Refs
Sprague-Dawley rat	Young	3 days	60% fat	Decreased GIR, increased HGP	Increased hepatic TG content	21
Sprague-Dawley rat	Adult	3 days	60% fat	Increased HGP	Increased hepatic TG content, increased PKCε and JNK activity in the liver	22
C57BL/6 mouse	12 weeks	4 days	45% fat	Decreased GIR	Glucose intolerance in GTT, increased hepatic lipid content, decreased TNFα mRNA in the liver	23
C57BL/6 mouse	5 weeks	3 days	60% fat	Decreased GIR, increased HGP	Increased body weight, epididymal adipose tissue weight, and hepatic lipid content, increased F4/80, CD68, CD11b and CD11c mRNA content in the liver	24
